# Removal of 4-Nitrophenol from Aqueous Solution by Using Polyphenylsulfone-Based Blend Membranes: Characterization and Performance

**DOI:** 10.3390/membranes11030171

**Published:** 2021-02-27

**Authors:** Ali Amer Yahya, Khalid T. Rashid, Maryam Y. Ghadhban, Noor Edin Mousa, Hasan Shaker Majdi, Issam K. Salih, Qusay F. Alsalhy

**Affiliations:** 1 Membrane Technology Research Unit, Chemical Engineering Department, University of Technology, Alsinaa Street 52, Baghdad 10066, Iraq; ali.a.yahya@uotechnology.edu.iq (A.A.Y.); 80007@uotechnology.edu.iq (K.T.R.); 80209@uotechnology.edu.iq (M.Y.G.); noor.e.mousa@uotechnology.edu.iq (N.E.M.); 2 Department of Chemical Engineering and Petroleum Industries, AlMustaqbal University College, Babylon 51001, Iraq; hasanshker1@gmail.com (H.S.M.); Dr_IssamKamil@mustaqbal-college.edu.iq (I.K.S.)

**Keywords:** 4-Nitrophenol, wastewater treatment, nanofiltration membrane, water quality, optimization, environment

## Abstract

Among many contaminants in wastewater, organic phenol compounds presented a major concern to endanger the water resources safety. In the present study, blend nanofiltration (NF) membranes comprising polyphenylsulfone (PPSU) and polyethersulfone (PES) were prepared via the non-induced phase separation and their performance was examined against 4-Nitrophenol (4-NP). The PES ratio in the dope solution was varied from 6 to 9 wt.% to probe the impact of PES on the retention and permeation characteristics of the final membranes. A series of experimental tools were employed to estimate the characteristics of the membranes, including surface and cross-section, hydrophilicity, pore size and pore size distribution. Performance evaluation of the NF membranes was conducted considering two operational variables; pH and initial feed solution. About 99% removal of 4-NP along with 6.2 L/m^2^·h·bar was achieved at the optimum operating conditions as revealed by optimization and mathematical modelling.

## 1. Introduction

As a multipurpose filtration and cost-effective process, nanofiltration (NF) membrane technology has exceedingly harnessed for water purification within the last few decades. This exceptional process was characterized by low operating pressure and high retention characteristics since the process merges both size and charge exclusion. With one-thousand molecular weight cut-off, the NF application spectrum was extended from concentrating and eliminating of versatile organic compounds presented in water and wastewaters to softening and removing of disinfection by-products [1,2]. In this context, nitrophenols are reckoned as one of the deleterious compounds that hardly could be taken away from aquatic systems. To date, unlike techniques have been harnessed for these contaminations removal from aqueous solutions. This includes; solvent extraction [3], adsorption [4], oxidation [5], ion exchange [6], biodegradation [7], reduction [8], electrochemical processes [9], photodegradation [10], pressure-driven membrane process [11], liquid membranes [12,13] and hybrid methods [14]. However, amongst these available techniques, NF process could endow an idealistic separation potency for organic compounds like 4-Nitrophenol. One of the most advantages of the NF membrane compare to other membrane separation processes such as RO and UF membranes is that it give ultra-higher mass production than RO and provides better solute rejection than UF at the same transmembrane pressure. The NF membrane was mainly applied in purification of water to separate organics with low molecular weight and salts for desalination of water. Based on the requirement of water quality, NF membranes can be layed at the end of the water purification process for water production process or before to the RO unit if quality of drinkable water is desired [11].

4-Nitrophenol is a well-known phenolic compound having two functional groups, NO_2_ and OH group located on the benzene ring. It has been employed across wide industrial sectors, such as drugs production (e.g., acetaminophen, aspirin), synthetic dyes, explosives, pharmaceuticals and to darken leather [15]. Nevertheless, it is a leading compound for fungicides, pesticides and insecticides production [16]. As a result of these huge manufacturing and processing, 4-Nitrophenol step inside the environmental system as organic contaminants. Ultimately, induce high toxicity for many living creatures, either directly or indirectly through some of their catabolic metabolites. Along with considering 4-Nitrophenol as one of the substantial xenobiotics in the groundwater and surface water resources, it could be formed in the atmosphere by OH-initiated photo-oxidation of aromatic hydrocarbons and broadly diffused in water and soil [17]. Commonly known, 4-Nitrophenol is highly soluble and stable in water bodies such as freshwater, marine environments and industrial wastewaters with a moderate release of acidity in water as a result of dissociation. The typical concentrations in wastewater discharged by chemical/organic industries are about 190 mg/L [18].

Presence of this compound imparts undesirable odour and taste to drinking water. Therefore, wastewaters bearing 4-Nitrophenol compounds must be treated adequately to meet the discharge quality criteria before being allowed to join external water bodies and should be kept at concentrations as low as 0.005 mg/L [19]. The U.S. Environmental Protection Agency (EPA) had listed 4-nitrophenol to its Emergency Planning and Community Right-to-know Act (EPCRA). This act has involved numerous hazardous and toxic substances while recommended that its concentrations in the natural and drinking waters should not exceed the 10 µg/L limits [20]. Besides that, its monthly average concentration in industrial effluents should not go further than 162 μg/L [21]. In this contest, acute exposure to 4-NP may in due blood disorders, kidney and liver damage, skin and eye irritation, anaemia and systemic poisoning [22]. Hence, the persistence of a compound with such high toxicity in the environment necessitates knowing the fate of these compounds and building up tendency. This is a primary goal in order to harness the adequate technologies for rapid remediation or detoxification of these compounds.

There are several NF membranes systems proposed for removal of 4-nitrophenol from aqueous solution, such as, Cellulose acetate (CA)/Triton [23], polysulfone (PS)/Ascorbic acid and (PS)/Citric acid [24], most of them are not polymer blends membranes. Nowadays, it was found in literature that PES has a favored hydrophilic character as well as ability of removal of various organic pollutants [25]. PPSU is one of the most favor polymers because of the great performance properties, good resistance against environmental effect, and thermal stability. From other side, rare works have been achieved utilizing PPSU membranes for various applications because of the hydrophobic character, therefore, PPSU membranes are significantly subject to fouling by organics pollutants.

The incoming concept of the current study is in the fabrication of PPSU-based blend membranes to greatly enhance the mass flux production and removal efficiency of the pollutants.

In the current work, an endeavour was made to probe the characteristics of two polymers merged to obtain a new class of membrane material. Polyethersulfone (PES) and polyphenylsulfone (PPSU) blend membranes have been prepared and expected to impart better performance and mechanical properties than the membrane composed of individual polymers [26]. To the best of the author’s knowledge, rare study has been conducted to investigate the influence of blend polymers on NF membrane characteristics. 4-Nitrophenol was chosen as foulant model to evaluate the membrane performance. So far, the removal efficiency of 4-Nitrophenol has not reached the required level as it has been found in the literature [23,24]. In addition, the mass production of the treated water found in the literature was lack; therefore, this lack in flow rate production was taking into account in this study. Alongside, tests were carried out under unlike conditions of feed concentration and pH while optimum conditions were employed for prolonged operations.

## 2. Experimental

### 2.1. Materials

**Figure 1:**
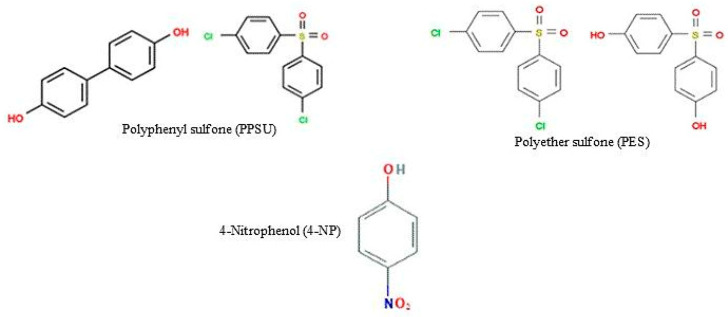
Chemical structures of PPSU, PES and 4-NP.

Polyphenylsulfone (PPSU, MW = 400.45 g/mol) supplied by Solvay Advanced Polymers (Brussels, Belgium) and Polyethersulfone (PES, MW = 232.26 g/mol) supplied by Sigma-Aldrich (St. Louis, MO, USA) was selected as the membrane material. Dimethyl sulfoxide (DMSO) was purchased from Sigma-Aldrich (St. Louis, MO, USA) and utilized as a solvent. 4-Nitrophenol (C_6_H_5_NO_3_, 99% purity) was used as received from Sigma-Aldrich (Shanghai, China) and Sodium hydroxide (NaOH, 97% purity) was obtained from (Shanghai, China). All chemicals were used as received without further purification. The chemical structure of PPSU, PES and 4-nitrophenol are given in Figure 1.

### 2.2. Experimental procedure

#### 2.2.1. Preparation of PPSU/PES Membranes

**Figure 2:**
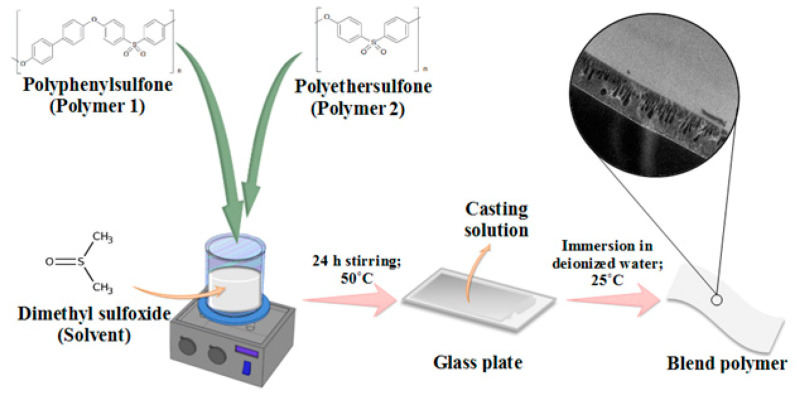
Schematic diagram for membranes preparation.

Five blend membranes were prepared via the classical phase separation technique Figure 2). Composition of the membranes was given in Table 1. Briefly, a 20% wt.% of PPSU and a certain amount of PES were dissolved in 80 wt.% DMSO and mechanically stirred overnight until a homogeneous casting solution was obtained. Following that, a proper amount was sprinkled on the glass plate and cast with a clearance gap of 200 µm. Thereafter, the prepared membrane was immersed in a deionized water bath to finalize the phase inversion process. The fabricated membranes were repeatedly rinsed with DI water and stored until further use. More details were presented elsewhere [27,28]

#### 2.2.2. Membranes Testing Rig Setup

**Figure 3:**
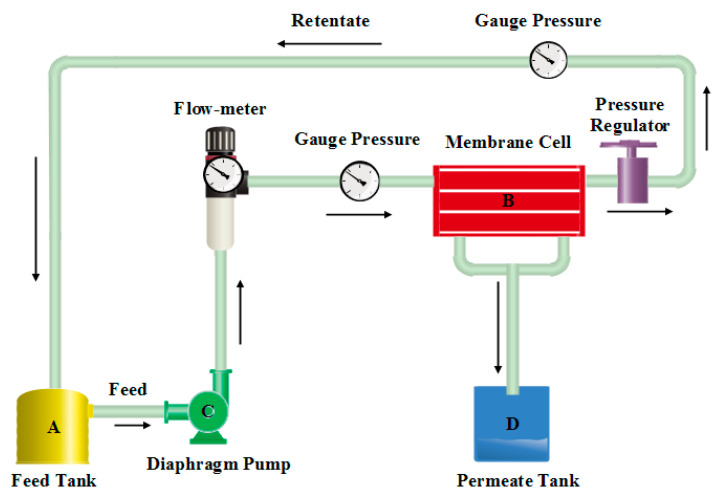
Schematic diagram of the experimental nanofiltration system.

All NF membrane flux and retention tests were conducted utilizing a custom-made cross-flow filtration set up with an effective membrane area of 16 cm^2^ at a low feed flow rate (1.4 L/min) and low operating pressure (3 bar). Figure 3 describes the schematic diagram of the experimental which equipped with a feed tank having a capacity of 1 L, membrane module, flowmeter and pressure gauges. The feed solution was fed through the membrane module by a diaphragm pump (working pressure =100 psi and flow rate of 25.4 GPH).

For flux and rejection experiments, the prepared membranes were cut into the desired size, placed inside the membrane module and finally compacted before taking the measurements [29,30]. The pure water permeability (*PWP*) was calculated using the Equation (1) below.

(1) $$PWP=A_{w}=\frac{Q_{w}}{\Delta P\times A_{S}}$$

where *A_w_* is the pure water flux of membrane (L/m^2^ h bar), *Q_w_* the permeate flow rate (L/h), Δ*P* is the pressure difference between the feed side and the permeate side of the membrane (bar), and *A_s_* is the effective membrane’s surface area (m^2^).

Following the pure water experiment, the tank was emptied and refilled with the 4-NP solution. Performance of the membranes was investigated in terms of both flux and rejection. To probe the influence of experimental parameters on the 4-NP removal, synthetic aqueous solution at initial concentrations (10^−5^, 10^−4^, and 10^−3^ M), and pH range from 6 to 14 were utilized. Permeance (*A_w_*) was estimated by using Equation (1) while 4-NP retention (*R*%) was based on Equation (2).

(2) $$R\left(\%\right)=\left(\frac{C_{F}-C_{P}}{C_{F}}\times 100\right)\;$$

where *C_F_* and *C_P_* were the concentration of 4-NP in the feed and the permeate solutions, respectively [31,32].

In order to detect the content of 4-NP, the absorbance was measured by UV–vis spectrophotometer (Chrom Tech UV-1100) at 318nm wavelengths.

### 2.3. Membranes Characteristics

The structural morphology of the fabricated membranes was inspected under a scanning electron microscope (TESCAN VEGA3LM, Oxford Instruments) (TESCAN VEGA3 SB, Kohoutovice, Czech Republic) to observe the top and cross-sectional morphologies of prepared membranes. The membranes samples were initially broken in liquid nitrogen fractured and sputtered with gold before imaging with the microscope at 10 kV.

Sessile drop method was employed to determine the static contact angle (CA) between distilled water and top surface of the membrane utilizing a contact angle measuring device (CAM 110, Taiwan).

The membranes porosity was calculated by using Equation (3) [33,34], while the mean pore radius (*r_m_*) of was estimated by Guerout-Elford-Ferry Equation (4) below:

(3) $$\varepsilon \;\left(\%\right)=\left(1-\frac{\rho_{m}}{\rho_{p}}\right)\;\;$$

where *ρ_p_* and *ρ_m_*, (g·cm^−3^) are the density of the polymer blend and membrane density, respectively.

(4) $$r_{m}=\sqrt{\frac{\left(2.9-1.75\varepsilon \right)8\eta \times l\times Q_{W}}{\varepsilon \times A_{S}\times \Delta P}}$$

where: *ε*, *η*, *l*, *Q_W_*, *A_S_*, and Δ*P* represented the porosity, water viscosity (Pa.s), thickness of the membrane (m), flow rate of the permeate (m^3^/s), effective area of the membrane (m^2^), and the operational pressure (0.3 MPa), respectively.

## 3. Results and Discussion

### 3.1. Impact of PES Content on the Structural Morphology

**Figure 4:**
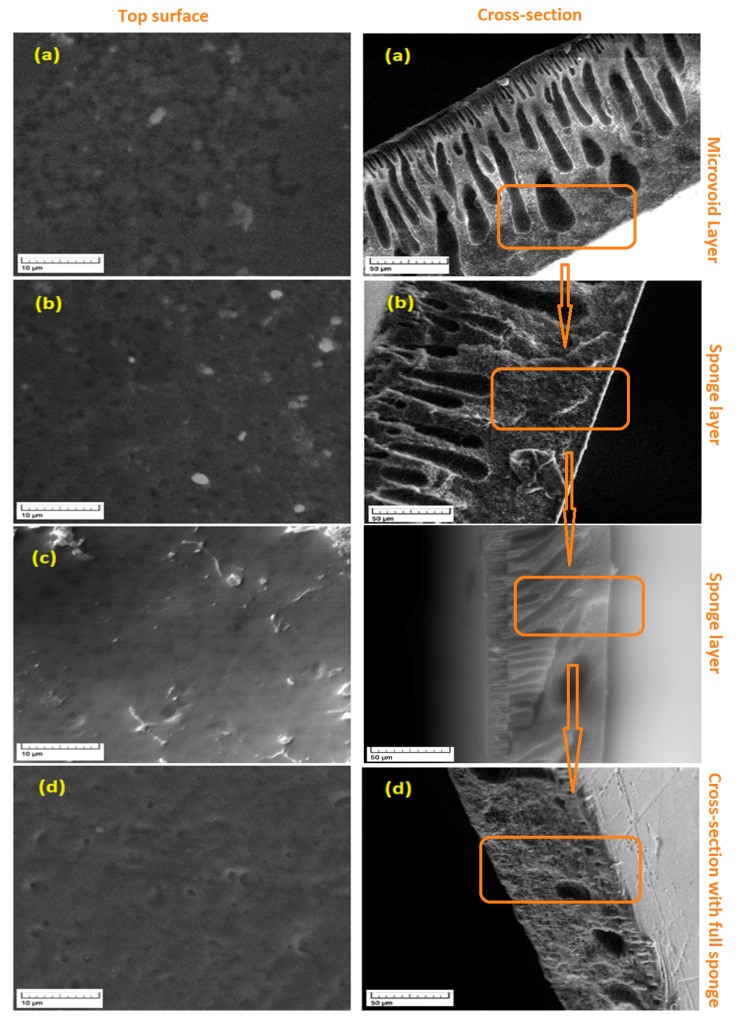
SEM images of cross-section and top surface of the membranes (**a**) PPSU_20_/PSU_0_, (**b**) PPSU_20_/PSU_6_ (**c**) PPSU_20_/PSU_7_, (**d**) PPSU_20_/PSU_9_.

One of the critical performance determinants in the preparation of the membrane is the introducing of an extra polymer in the casting mixture aiming for modifying the structure of the membrane and, in turn, performance of the membrane [35]. In the present work, the impact of the PES content on the PPSU membrane structure was studied utilizing five PES amounts (0, 6, 7, 8, and 9 wt.%). The SEM images for both cross-section and top surface of the resultant blend membranes were shown in Figure 4.

As can be noticed in Figure 4a, the membrane fabricated from only PPSU (20 wt.%) had a finger-like structure at the top with a randomly distributed macro-voids at the bottom. The walls of the macro-voids were thick with closed ends. Also, a sponge layer had taken place as an important layer of the cross-section. This was due to the relatively high viscosity of dope mixture caused by the high polymer concentration used. The addition of 6 wt.% of PES into the dope solution had enhanced the membrane structure (Figure 4b). The macro-voids have been disappeared and a well-formed finger-like structure was noticed with a sponge structure at the bottom. An additional amount of PES (up to 7 wt.% in the casting mixture) led to an increased solubility parameter between the polymer blend and solvent and resulted in a more open porous structure (Figure 4c). Higher additive rates (8wt.% and 9wt.%) induced higher solution viscosity and the lower diffusion rate between the solvent and nonsolvent. Hence, the properties of the membrane were affected to end up with a denser structure and full sponge cross section structure (Figure 4d). It was observed that slowly dissolved of the PPSU in the solvent but manifested a faster rate in presence of PES (7wt.%) whereas decreased again after adding a higher amount of PES (8, and 9 wt.%).

**Figure 5:**
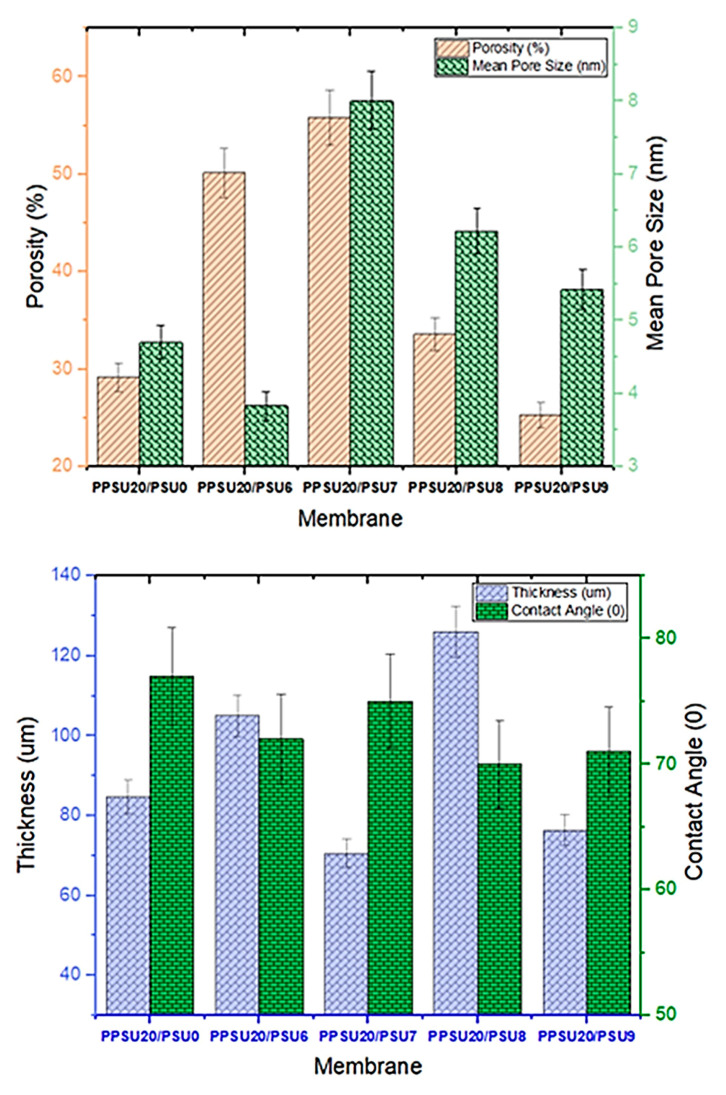
Characteristics of the membranes. Porosity and mean pore size (**Top**), and membrane thickness and CA (**Bottom**).

Nevertheless, the effect of PES addition was explicit on the membranes porosity, however, this effect was trivial and did not follow any trend for the mean pore size measurements (Figure 5, Top). Comparing to the lowest witnessed porosity (29.17%) of the control PPUS membrane, the membrane prepared with 6 wt.% and 7 wt.% PES has manifested an increased porosity value to about 50.13% and 55.81%, respectively. However, further addition of PES up to 8 wt.% and 9 wt.% has led to a decline in the porosity to 33.60% and 25.29%, respectively. This phenomenon was attributed to the reduction in solubility parameter difference between the polymer mixture and NMP solvent with increasing of PES as reported in the literature [23]. This means that the chains of PES have lower impact on the NMP solvent than the PPSU, therefore fast rate of diffusion of NMP and water could take place, which in turn results to membrane with high porosity and pore size.

Determining the hydrophilicity/hydrophobicity of a membrane surface could provide an indication for the membrane fouling behaviour. Impregnating the PES within the PPSU polymeric matrix at various ratios did not showcase any distinguished trend for contact angle measurements. Pure PPSU membrane has manifested the highest contact angle (77) if compared with other blend membranes which displayed 72, 75, 70 and 71 for the 6 wt%, 7 wt%, 8 wt% and 9 wt%, respectively (Figure 5, Bottom). All results were summarized in (Table 2) below.

### 3.2. Role of Operational Conditions on the Performance of Blend Membranes

**Figure 6:**
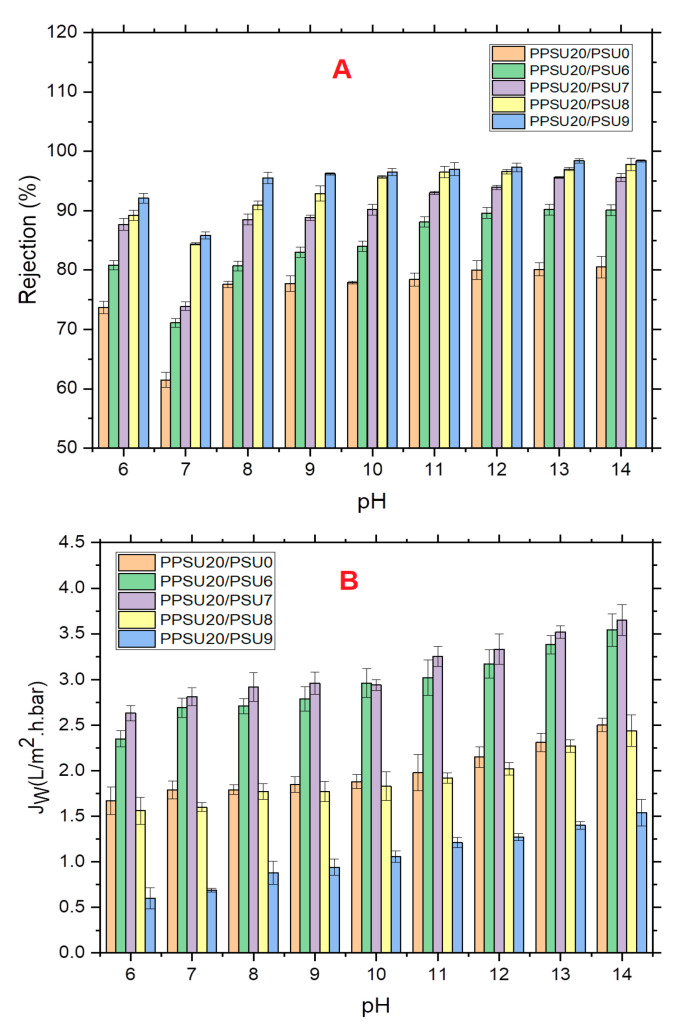
Blend membranes rejection (**A**) and permeation (**B**) using 10^−4^ M 4-NP (1 h, 25 °C and 3 bar).

The influence of pH value of the feed mixture on the retention/permeation characteristics of NF blend membranes has been probed against 4-NP as an organic model. All membrane results of the rejection and permeation against a basic range of pH were depicted in Figure 6.

**Figure 7:**
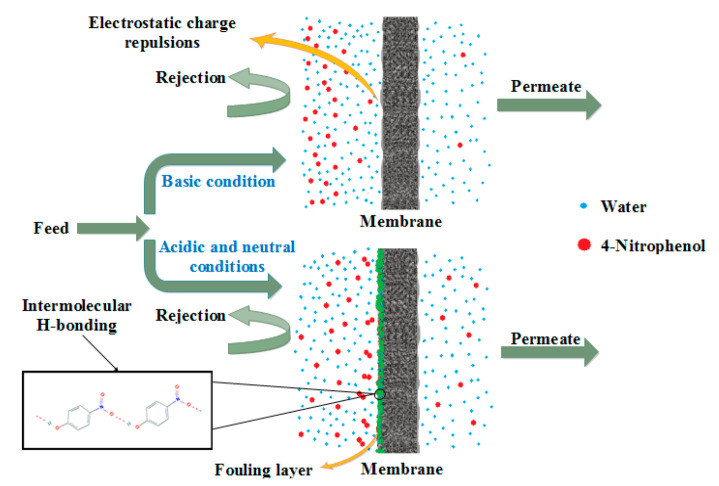
Impact of the pH conditions on the permeation and retention characteristics of the membranes.

The present study focuses on the rejection of 4-NP at basic pH values, as shown in Figure 6A. Unsurprisingly, retention of all blend membranes manifested an increasing trend with racing the pH values. This was ascribed to the fact that at the alkalinity conditions, both membranes and 4-NP molecules are negatively charged. Herein, strong electrostatic charge repulsions would take place to hinder 4-NP molecules deposition on the membrane’s surface. Thus, the membrane would experience a higher rejection performance. In the meantime, increasing the PES content within the PPSU membrane matrix resulted in higher rejection capabilities. For instance, blend membrane with 9%PES experienced 98% 4-NP removal at pH14 comparing to only 81% removal for nascent PPSU at the identical operational conditions. Meanwhile, solute permeation showcased an increasing trend with increasing the pH values for all membranes. However, at certain pH values, the blend membranes permeation behaved differently depending on their porous structure (Figure 6B). Permeation of solutions containing 4-NP molecules manifested an increase by the addition of PES up to 7% and then decreased afterwards. The unusual peculiarities of high water flux for the membrane fabricated from 7 wt.% of PES as a polymer blend was attributed to the porous membrane structure resulted from the optimum exchange rate between polymer blend solution and water. Solubility parameter difference between the polymer mixture and NMP solvent decreased with increasing of PES as reported by Ghadhban et al., [26]. This means that the chains of PES have lower impact on the NMP solvent than the PPSU, it means that fast rate of diffusion of NMP and water, which in turn results to membrane with high porosity and pore size. It should be noted here that separation of organic solutes, such as 4-NP, by NF membranes could be a complicated process. The separation could be influenced by several mechanisms e.g., electrostatic charge repulsion and adsorption phenomena and not only limited to size exclusion. At some circumstances, electrostatic charge interactions could endow a higher retention against small charged organic pollutant if compared with the effects of size exclusion. From the other side, adsorption of organic components with hydrophobic character could be significant during the filtration of ionizable components when they are electrostatically neutral and ultimately solute permeation tend to decrease significantly. Herein, 4-NPs have revealed increasing retention behaviour at the pH range used because of their acid dissociation constant (*pK_a_* = 7.15) [36]. This implies that, if *pK_a_* was greater than solution pH, 4-NP molecules would be presented in both forms; ionized (4-NPO^−^) and un-ionized (4-NP) species. Whereas in case of *pK_a_* was smaller to solution pH, all 4-NP molecules would be only as ionized (4-NPO^−^) species. In acidic and neutral conditions, 4-NP molecules could have less negative charges implying that just few of these molecules are ionized and have negative charges. Whereas, all 4-NP molecules in basic solutions possess only negative net charges because of the complete ionization of the phenolic hydroxyl group to yield the phenolate ion [23]. The influence of the PPSU/PES ratio on the permeation and separation performance of the membranes under various pH conditions were illustrated in Figure 7, below.

**Figure 8:**
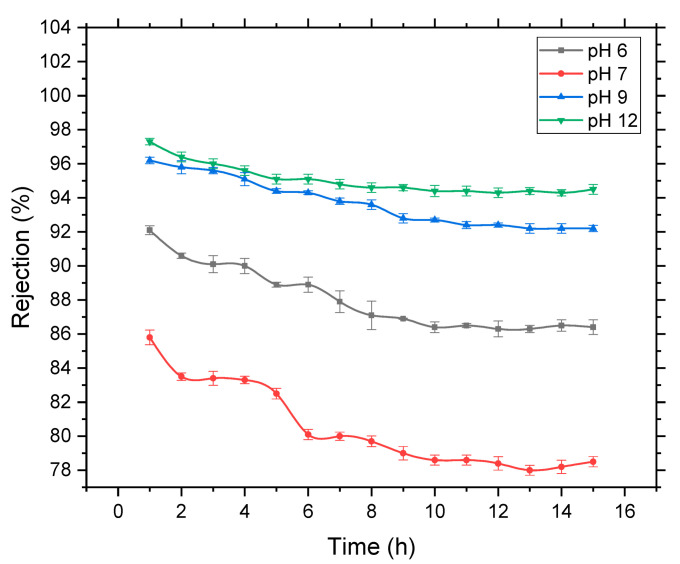
Rejection of 10^−4^ M 4-NP solution versus time using PPSU_20_/PES_9_ membrane at various solution pH, 25 °C, and 3 bar.

Worth mentioning, during the prolonged operation, rejection of 4-NP molecules has declined with time and reached a constant value. This has indicated that a layer of 4-NP was formed at the surface of the membrane. However, the drop in the rejection of 4-NP was varying depending on the pH solution condition (Figure 8). The observed decline in the retention of 4-NP molecules was trivial at basic solution comparing to the acidic and neutral conditions. This could be described depend on the dominant mechanism contributing to the retention of 4-NP molecules. Within alkaline conditions, electrostatic repulsion between both negatively charged membrane surface and 4-NP molecules were initially crucial for the removal of the solutes. Hence, retention values did not change noticeably over time because of unpretentious adsorption of 4-NP on the surface of blend membrane. However, in acidic conditions, the retention of 4-NP declined as the time passes due to rapid deposition of solutes and the formation of intermolecular hydrogen bonds on the surface of the membrane. This decline in retention continues to reach the steady-state conditions according to the conditions of the solution.

**Figure 9:**
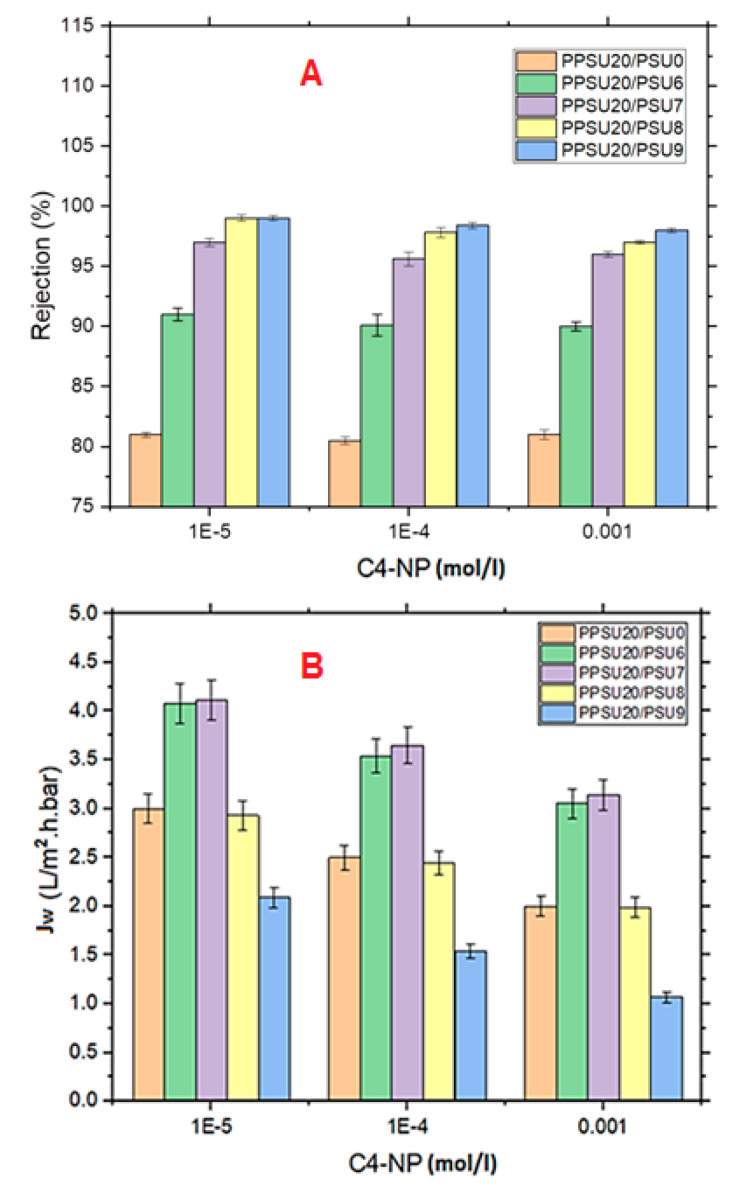
Rejection (**A**) and permeation (**B**) of 4-NP at different feed concentrations (pH 14, 25 °C and 3 bar).

The impact of initial composition of the feed on rejection and permeation flux of 4-NP was illustrated in Figure 9. A nominal variation in the retention values was barely obtained with the increasing of the initial feed composition in Figure 9A. However, with the increasing the initial feed concentration, a slight decline in the permeate flux values were observed for all membranes Figure 9B. This indicated that adsorption of hydrophobic organic molecules occurred at the membrane surface, especially at higher feed compositions. Depending on the solute concentration used, a cake layer was formed at the surface of the membrane along with rapid pore blockage and prevented the water passage through the membrane. Higher solute concentration induced faster internal and external fouling of the membranes and higher flux decline, indeed. Similar behaviour was reported in previous works of literature [24,37].

### 3.3. Comparison Study

Table 3 depicts a comparison between the retention of 4-NP from aqueous solutions performed by the NF membranes prepared in the current work with the removal efficiency of NF membranes selected from the literature. The most important characteristic of the membranes such as polymer material and operating conditions such as 4-NP composition, pH value and the transmembrane pressure were also depicted in Table 3. It seems that the removal efficiency of the membranes has a high value in comparison with most NF membranes selected from the published works.

### 3.4. Optimization and Modeling

To further understand the contribution of the involved operating parameters and characteristics on the performance of the blend membranes, optimization and mathematical modelling was conducted. This had a pivotal importance in knowing the whole system response to the operational parameters with the available membrane features to bestow a better understanding of the entire process. The developed optimization design for membrane permeates flux response was verified statistically through the analysis of variance technique (ANOVA). This analysis has presented a good coefficient for the determination value.

ANOVA is a combination of statistical models harnessed to check the variations between two or more parameters. More precisely, it has been employed to assess the influence of the independent parameters on the dependent variables through regression analysis. This technique can estimate the optimum conditions of process operating variables more strictly via testing the relative importance of the variables. ANOVA was carried out with the aid of a software package (MINITAB^17^) for a level of significance of 5%. Table 4 listed the ANOVA results for the membrane rejection and permeate flux data.

**Figure 10:**
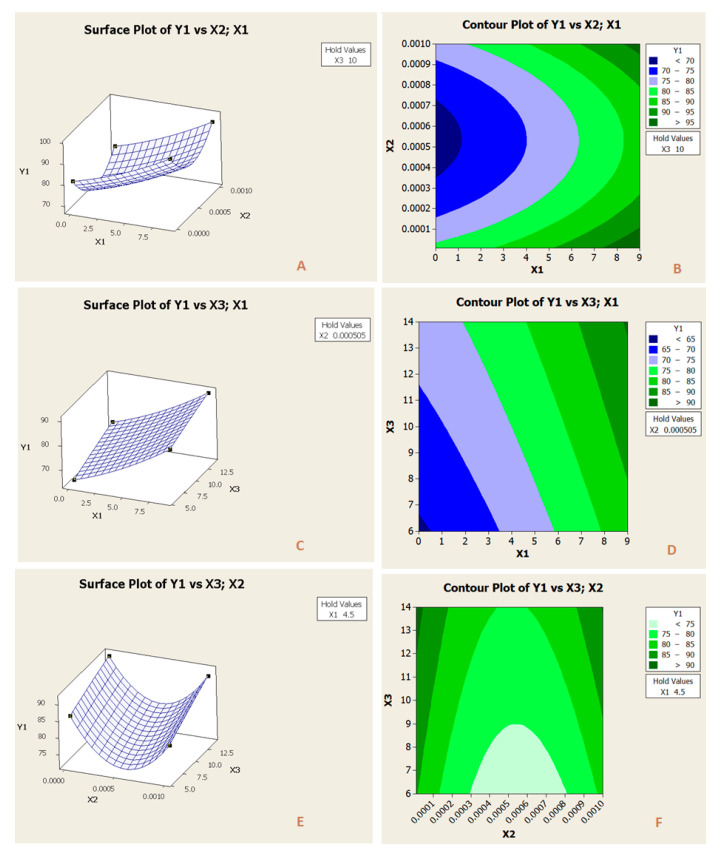
Surface plot (**A**,**C**,**E**), and contour plot (**B**,**D**,**F**) of the parameters impacts (PES concentrations (x_1_), feed concentrations (x_2_) and pH (x_3_)) on rejection.

**Figure 11:**
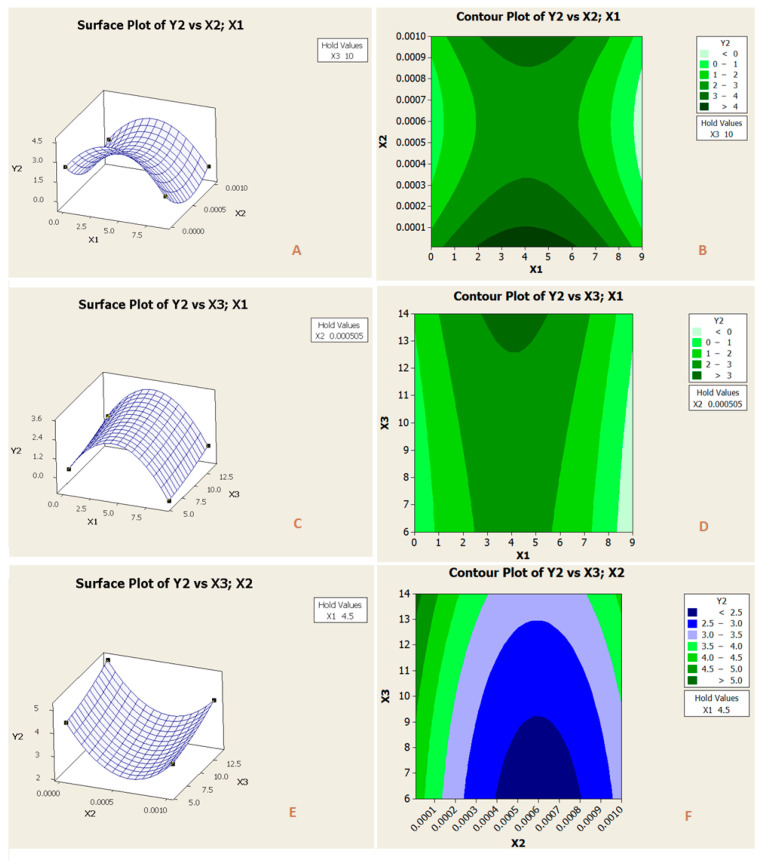
Surface plot (**A**,**C**,**E**), and contour plot (**B**,**D**,**F**) of the parameters impacts (PES concentrations (x_1_), Figure 2. and pH (x_3_)) on permeate flux.

Figure 10 and Figure 11 illustrated the surface and contour plots of the standardized impacts of rejection and permeate flux, respectively. It can be inferred clearly that the feed concentration, pH, and characteristics of the membranes were pivotal and played an essential role during the operation. Figure 10A,B, presented the effect of the two factors, PES concentrations, x_1_ and feed concentrations, x_2_, when the third-factor acidity measure, x_3_ was held at 10. As it can be noticed in the figure, the rise of PPES concentration up to 9 wt.% led to an increase of retention coefficient (y_1_) up to 98%, while the variation of feed concentration had a less significant influence on retention coefficient.

The response surface plot and contour plot of the retention coefficient response function (Figure 10C,D) revealed that increasing PES concentrations, x_1_, and decreasing pH, x_3_, resulted in higher retention coefficients at constant feed concentration x_2_ = 5.05 × 10^−4^ M, while the effect of variation of PES concentrations value on retention coefficient was insignificant for lower values of pH.

In Figure 10E,F, the effect of feed concentration, x_2_ and pH, x_3_ is depicted at constant PES concentration, x_1_ = 4.5. It is observed that decreasing feed concentration and decreasing pH resulted in a higher rejection of 4-NP. And again, the highest values of retention were obtained at the less of feed concentration.

In Figure 11A,B, the effect of two factors, x_1_ (PES concentrations) and x_2_ (concentration), when the third factor x_3_ (pH) was held at (10) is presented. As it can be observed from the figure, the rise of PPES concentration up to 7 wt.% led to increasing of permeate, y_2_, up to 4 L/m^2^·h·bar, while the variation of feed concentration had an high significant influence on permeance.

In Figure 11C,D, the effect of PES concentrations, x_1_, and pH, x_3_ is depicted at constant feed concentration, x_2_ = 5.05 × 10^−4^ M. It is observed that increasing pH resulted in the higher permeance of 4-NP. And again, the highest values of permeance were obtained in the middle region of PES concentration.

In Figure 11E,F, the effect of two factors, x_2_ (feed concentrations) and x_3_ (pH), when the third factor x_1_ (PES concentrations) was held at (4.5 wt.%) is presented. As it can be observed from the figure, the decline of feed concentration led to an increase of permeance, while the variation of feed concentration had a very high significant influence on permeance.

**Figure 12:**
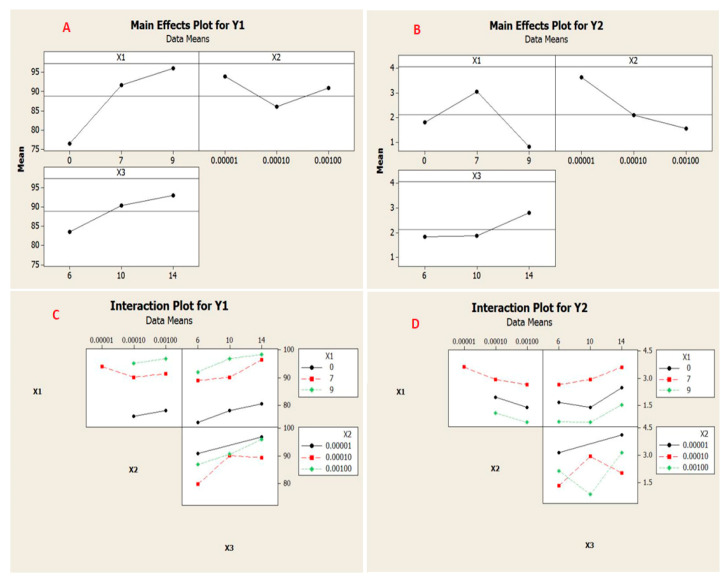
Major impact plot for rejection (**A**) and membranes permeate flux (**B**), and interaction plots for rejection (**C**) and permeate flux (**D**).

Figure 12A,B clarified the mean impact of rejection and flux. As can be observed, the maximum retention was acquired when the PES ratio within the PPSU membrane matrix was 9wt.%, feed concentration was 10^−5^ M, and at pH solution equivalent to 14. In this context, the permeate flux was found to at its maximum value when the PES ratio was 7 wt.%, feed concentration was10^−5^ M, and pH solution was 14.

The interactions results between adopted parameters were shown in Figure 12C,D. The lines represented the PES%, pH, and concentration of the feed. Therefore, it has been pointed out that there was considerable interactions impact occurred amongst the adopted parameters.

**Figure 13:**
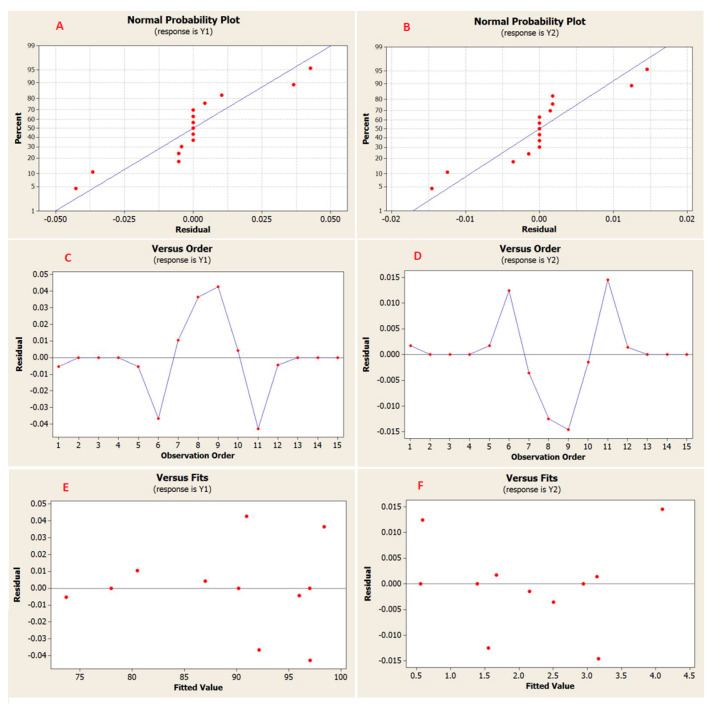
Normal probability plot of residuals for rejection (**A**) and permeate flux (**B**), Plot of residuals with data order for rejection (**C**) and permeate flux (**D**), and The residuals versus fitted values Plot for rejection (**E**) and permeate flux (**F**).

Figure 13A,B showcased the normal probability plot. The normal probability plot is a diagrammatic method to estimate whether the data collection was normally distributed or not. Data was plotted against theoretical normal distribution in a mode where the points fall along the straight line. Points away from the straight line could be neglected since the values considered as not true. From the plotted data, it can be realised that all data residuals were distributed normally since the overall points have formed an approximate straight line. The parameters have affected the response “Outliers don’t exist in the data”.

The residual graph was introduced to examine the goodness-of-fit in ANOVA analysis Figure 13B,C. The graph has displayed the independent parameters on the x-axis whereas the residuals were on the y-axis. It can infer the crucial assumptions about the errors: The errors were independent of each other and the better method to gain an independent error was through ordering the run of experimental trials randomly. The errors difference does not alteration for the various level of parameters. It also evident that the difference of the errors does not change according to the values of the expected response, clarified in Figure 13E,F.

## 4. Conclusions

PPSU-based blend NF membranes of various PES contents as additive were prepared for removal of 4-nitrophenol from aqueous solutions. From SEM images it can be conclude that the addition of PES results to change the structure of PPSU-based blend membranes from a finger structure to a fully sponge structure. Higher mean pore size (8.01 nm) was recorded at 7 wt.% PES due to the porous membrane structure resulted from the optimum exchange rate between polymer blend solution and water (reduction in solubility parameter difference between the polymer mixture and NMP solvent). The 7 wt.% showcased highest porosity (55.8%) comparing to control PPSU membrane (29.1%) and other blend membranes with higher PES%. Even though there was a change in the hydrophilicity of modified membranes but it was trivial compared to the control membrane. Moreover, blend membranes with 9 wt.% revealed higher retention of 4-nitrophenol (98.4% at pH14) at all pHs among other membranes due to their greater dense structure. In the meantime, the solute permeation manifested a similar increasing trend against the pH value. Membrane prepared with 7 wt.% revealed higher permeation (4.11 LMH) characteristics among control and other PPSU-PES blend membranes. Optimization and mathematical modelling were conducted to estimate the optimum operation parameters utilizing ANOVA analysis. Results demonstrated that the best rejection results were obtained when operating conditions found to be for feed concentration of 10^−5^ M, pH solution of 14, and 9 wt.% PES where a maximum value (99%) of the 4-NP rejection was obtained. In the other hand, gained optimum operating conditions were found to be 10^−5^ M for the feed concentration, pH solution of 14, and 7 wt.% PES for the maximum value of the 4-NP permeance (4.11 L/m^2^·h·bar).

## Figures and Tables

**Table 1:** Compositions of PPSU/PSU blend solutions and PWP of the membranes.

Membrane Code	Casting Solution Compositions (wt%)		Solvents (NMP) (wt%)
	PPSU	PES	
PPSU_20_/PSU_0_	20	0	80
PPSU_20_/PSU_6_	20	6	74
PPSU_20_/PSU_7_	20	7	73
PPSU_20_/PSU_8_	20	8	72
PPSU_20_/PSU_9_	20	9	71

**Table 2:** Effect of addition of different concentrations of PES on thickness, and water permeability of the PPSU membranes.

Membrane Code	Thickness (μm)	PWP (L/m^2^ h bar)
PPSU_20_/PSU_0_	84.64 ± 1.13	1.612 ± 0.28
PPSU_20_/PSU_6_	104.97 ± 1.05	1.752 ± 0.05
PPSU_20_/PSU_7_	70.50 ± 0.11	13.369 ± 0.11
PPSU_20_/PSU_8_	126.00 ± 18.24	2.259 ± 0.21
PPSU_20_/PSU_9_	76.35 ± 0.24	2.004 ± 0.18

**Table 3:** Compare the results of 4-NP rejection of blend membranes against preceding literature.

Process	Type	Compositions (wt.%/wt.%)	C (mM)	pH	ΔP (bar)	R (%)	Permeation Flux (kg/m^2^·h)	Ref.
NF membrane	PPSU/PES	20/9	0.01	14	3	99	6.2	This work
NF membrane	PPSU/ PES	20/9	0.1	14	3	98	4.6	This work
NF membrane	PPSU/ PES	20/8	0.1	14	3	98	7.3	This work
NF membrane	PPSU/ PES	20/7	0.1	14	3	96	10.9	This work
NF membrane	PPSU/ PES	20/9	0.1	8	3	96	2.6	This work
NF membrane	PPSU/ PES	20/8	0.1	8	3	91	5.3	This work
NF membrane	PPSU/ PES	20/7	0.1	8	3	89	8.7	This work
NF membrane	CA/CTAB	17/0.45	0.1	8	4.5	89	4.3	[23]
NF membrane	CA/Triton	17/0.45	0.1	8	4.5	71	3.7	[23]
NF membrane	CA/SDS	17/0.45	0.1	8	4.5	91	5.1	[37]
NF membrane	PS/Ascorbic acid	18/1	0.1	8	4.6	89	9.2	[24]
NF membrane	PS/Citric acid	18/1	0.1	8	4.6	91	8.0	[24]
NF membrane	PS/Malic acid	18/1	0.1	8	4.6	90	8.6	[24]
NF membrane	CA/Palmitic acid	17/2	0.1	8	4.6	87	5.8	[26]
NF membrane	CA/Oleic acid	17/2	0.1	8	4.6	83	7.8	[26]
NF membrane	CA/Linoleic acid	17/2	0.1	8	4.6	83	8.1	[26]
Low-pressure reverse osmosis	Thin-film composite polyester (RO90 membrane)	Polyamide	0.7	8	15	97	50.8	[38]

**Table 4:** ANOVA Analysis.

No.	%PES (X1)	C, M (X2)	pH (X3)	R% (Y1)	A_W_, L/m^2^·h·bar (Y2)
1	0	1.00 × 10^−4^	6	73.7	1.67
2	9	1.00 × 10^−3^	10	97	0.56
3	0	1.00 × 10^−3^	10	78	1.39
4	9	1.00 × 10^−3^	10	97	0.56
5	0	1.00 × 10^−4^	6	73.7	1.67
6	9	1.00 × 10^−4^	6	92.1	0.60
7	0	1.00 × 10^−4^	14	80.5	2.50
8	9	1.00 × 10^−4^	14	98.4	1.54
9	7	1.00 × 10^−5^	6	91	3.15
10	7	1.00 × 10^−4^	6	87	2.15
11	7	1.00 × 10^−5^	14	97	4.11
12	7	1.00 × 10^−3^	14	96	3.14
13	7	1.00 × 10^−4^	10	90.2	2.94
14	7	1.00 × 10^−4^	10	90.2	2.94
15	7	1.00 × 10^−4^	10	90.2	2.94

## Data Availability

Not applicable.
